# Enhanced water treatment via combined oxidation and adsorption: a synergistic approach

**DOI:** 10.3389/fchem.2025.1747407

**Published:** 2026-01-05

**Authors:** Pan Shulin, Walid Tahri, Amani Khaskhoussi, Ahmed H. El-Sappah

**Affiliations:** 1 Key Laboratory of Yangtze Aquatic Environment, Research Base, Yibin University, Yibin, China; 2 Department of Engineering, University of Messina, Messina, Italy

**Keywords:** adsorption, advanced oxidation processes, biochar, micropollutants, water treatment

## Abstract

Organic micropollutants are still a major environmental and public health problem because they accumulate in all water source over the world. Because standard treatment is ineffective at the low concentrations at which these contaminants are present to remove, there is an urgent need to find better tertiary treatments for wastewater. The key points covered in this review are adsorption and Advanced Oxidation Processes (AOPs). The use of certain adsorption materials makes it possible to selectively sequestrate impurities, while in the case of AOPs destructive processes are performed by means of reactive radicals leading to mineralization of pollutants. These methods complement each other and provide both efficiency, waste reduction, and sustainability benefits-providing a synergy of their strengths. The AOP adsorption hybrids reported in this paper represent one of the emerging types of water-treatment units, which are particularly relevant given the high demand for improved tertiary treatment technologies capable of effectively removing persistent micropollutants from wastewater.

## 1 Introduction

A secure source of water is important for people’s health, the environment, and the economy. But there are a lot of other chemical pollutants that are putting the quality of the world’s freshwater supplies at risk (UNESCO, 2023; Kümmerer et al., 2018). One hallmark of the new age is the problem of “micropollutants” along with more conventional pollutants. These chemicals are persistent and physiologically active, thus even at low concentrations (ng/L to µg/L), they can create major ecological problems and adverse health impacts in humans (Patel et al., 2019; Yang et al., 2017). Pharmaceuticals and personal care products (PPCPs), per-and polyfluoroalkyl substances (PFAS), and endocrine-disrupting chemicals (EDCs) fall under this category (Al Amin et al., 2020; Zabrebska and Bajkacs, 2023). PFAS are synthetic organic chemicals with fluorine atoms connected to alkyl chains that make things water- and stain-resistant. They are also referred to as the “forever chemicals” because they prefer to remain accumulated for extended periods of time in the environment. Even while traditional wastewater treatment plants (WWTPs) are mainly meant to get rid of nutrients, pathogens, and bulk organic matter (BOD/COD), they are largely ineffective against micropollutants like these (Grandclément et al., 2017; Luo et al., 2014). Water treatment plants (WWTPs) serve as both treatment facilities and major point sources, continuously releasing bioactive compounds into rivers, lakes, and groundwater. Studies revealed that certain pharmaceuticals have extremely low clearance rates in standard WWTPs. For example, Carbamazepine elimination can be less than 10%, leaving more than 90% of the influent concentration unaltered (Okuda et al., 2008). Further, WWTP effluents are a significant source of pharmaceutical contamination, with typical removal efficiency of <30–40% (Chaber-Jarlachowicz et al., 2025). It is usual and hazardous for these pollutants to enter into water sources that people drink (Pal et al., 2010; Richardson and Kimura, 2020). Significant research efforts have been directed towards cutting-edge tertiary treatment unit procedures that can target these resistant molecules, because traditional treatment methods have not worked at all (Shannon et al., 2008).

Adsorption techniques and AOPs have been extensively researched and could revolutionize various industries. To put it simply, AOPs are fundamentally “destructive” technologies. They cannot operate without creating powerful reactive oxygen species (ROS), like the sulfate radical (SO_4_^−^) and the hydroxyl radical *OH. These radicals oxidize organic pollutants in an unselective manner, leading to their mineralization and the subsequent formation of harmless byproducts like carbon dioxide, water, and inorganic ions (Glaze et al., 1987; Vinayagam et al., 2024; Wang and Zhuan, 2020). Adsorption, on the other hand, is a physical technology that is “separation-based” and depends on the physisorption or chemisorption of contaminants from the liquid phase onto a solid sorbent material’s large surface in order to concentrate them for later disposal or destruction (An et al., 2016; Crini and Lichtfouse, 2019).

Research on AOPs and adsorption techniques has often been carried out separately. The possibility of combining the two technologies to attain synergistic performance, leading to treatment efficiencies higher than each method alone, is, nevertheless, becoming more apparent. This change demonstrates a growing comprehension that questions traditional, independent methods (Alatabe et al., 2024; Silva, 2025). For instance, adsorption has the ability to pre-concentrate diluted contaminants, which in turn makes the subsequent AOP treatment more efficient and kinetically beneficial. Regenerating old adsorbents with AOPs is the other way around; this turns a waste product into a resource again and completes the material cycle (Gkika et al., 2025; Zhai et al., 2023).

This mini-review aims to outline the current state of the art in AOPs and adsorption, specifically concentrating on the material breakthroughs and mechanistic improvements that have been reported in the recent 5 years. A newly-created and exhaustive table will be used as a guide for the critical comparison of their capacities. This review will center on combined AOP-adsorption systems, which are at the forefront of research into creating resilient and effective water treatment solutions. The immediate threat of micropollutant contamination is so what these technologies are intended to combat (Miklos et al., 2018; Oturan et al., 2018). In other way, this work discusses and highlights adsorption and AOPs as key approaches; yet, both still encounter significant drawbacks that limit their effectiveness in real-world applications, including selectivity, stability, and energy consumption. By combining their capabilities, we can see where they overlap and where there are significant technology gaps that need filling in order to develop treatments that are effective, scalable, and long-term viable.

## 2 Cutting edge destructive remediation: advanced oxidation processes

There has been a lot of progress in AOPs, which have gone from being quite simple systems to being very complex and efficient technologies. Scientific efforts have centered on improving the efficiency of radical generation, broadening the pH range that can be employed, and making better use of renewable energy sources. Particularly noteworthy is the fact that UV-LED integration has pushed the use of UV-based AOPs to new heights.

### 2.1 AOPs based on light: retrofitting mercury lamps to power LEDs

For activating oxidants such as hydrogen peroxide and persulfate, UV-LEDs present a more efficient and versatile option compared with conventional mercury lamps. These include a smaller footprint, better on/off response speed, and wavelength selectivity (Li X. et al., 2019; Zhi, 2024). The second new frontier is vacuum ultraviolet (VUV) techniques, which photolyze water directly to generate •OH, but their scalability is limited by energy cost and water quality (Darandale et al., 2023). Graphitic carbon nitride (g-C_t_N_4_) and bismuth oxyhalides (BiOX) are two emerging families of semiconductor material that are presently enhancing the efficiency of heterogeneous photocatalysis upon excitation by visible light (Ahmed et al., 2024; Wei et al., 2021). The development of S-scheme heterojunctions, which are more efficient in charge separation than regular Type-II heterojunctions, represents a significant step toward more efficient photocatalysis (Fang and Wang, 2023).

### 2.2 Activation by chemical means: advanced oxidation process: persulfate and systems similar to fenton

Researchers are currently concentrating on finding ways to activate persulfate (PS) so that it can form sulfate radicals (SO_4_^−^). Compared to •OH, SO_4_^−^ has a longer half-life, is more effective across a wider pH range, and is more selective for electron-rich compounds (Wang X.-L. et al., 2021; Wang and Wang, 2018). Graphene and nitrogen-doped biochar are two examples of metal-free PS/PMS activators that have recently attracted a lot of attention from researchers looking for ways to stop toxic metal leaching (Gao et al., 2022). While this was going on, conventional Fenton reactions were aided by heterogeneous Fenton-like systems. These approaches sidestep the issue of sludge formation that happens in the homogeneous Fenton process by utilizing bimetallic catalysts (such Fe-Cu or Fe-Mn) or solid iron oxides, which function at pH values around neutral (Li J. et al., 2019; Montoya-Bautista et al., 2019). A new level of mechanistic insight and the possibility of selective oxidation have been brought about by the investigation of non-radical routes, like the formation of singlet oxygen (´O_2_) or direct electron transfer in these systems (Gao et al., 2022). When the goal of treatment is to eliminate priority or persistent contaminants, selectivity can improve degradation efficiency, decrease chemical usage, and prevent unexpected transformations, among other practical benefits. On the other hand, actual wastewater matrices often contain a variety of micropollutants, therefore over selectivity could be an issue. For thorough removal in these situations, broad-spectrum reactivity is ideal, since highly selective materials might not work as well as expected.

There are significant limitations to each of the three types of AOPs light-driven, persulfate-activated, and Fenton-type despite their great degrading capabilities. When it comes to energy efficiency and mercury elimination, UV-LED systems are a good example; nevertheless, their scalability is limited due to issues with heat management and uneven light dispersion. The same is true for persulfate-based AOPs; they can function throughout a broader pH range, but they can be difficult to activate and can produce potentially dangerous intermediates. Fenton and similar processes, on the other hand, are straightforward and efficient, but they still have limitations such producing iron sludge and having very specific pH requirements. When all factors are considered, it is clear that no AOP is inherently better than any other. This highlights the need for ongoing research into methods to decrease by-products, enhance catalyst stability, and guarantee performance in real-water settings.

## 3 Developing state-of-the-art adsorption methods for targeted sequestration

Although activated carbon is still used as a standard, new sorbents that are sustainable, inexpensive, and tailored to remove particular contaminants are changing the face of the adsorption industry.

### 3.1 Carbonaceous materials: sustainable and engineered

As a more environmentally friendly substitute for activated carbon, biochar a solid rich in carbon that is produced by pyrolyzing biomass waste has become quite popular in recent years (García-Prats et al., 2024). To improve its surface chemistry and attract certain contaminants, such as phosphate, heavy metals, or even PFAS, “designer biochar” is modified after synthesis using acids, bases, or metal impregnation (Mulabagal et al., 2025; Sharma et al., 2025). The vast surface area and customizable surface chemistry of carbon nanotubes (CNTs) and graphene oxide (GO) are being studied for applications beyond biochar, but their prohibitive costs prevent their widespread use. Additionally, MXenes, such as Ti_3_C_2_T_x_, show promise as adsorbents, however they easily degrade in water due to oxidation. Strategies such as chemical passivation of reactive areas, surface-termination engineering, and encapsulation or composite in polymers or carbon can be employed to improve their stability. Although additional research is needed to determine the methods’ long-term stability in real-world wastewater circumstances, it has been demonstrated that these approaches can postpone oxidation and maintain functional performance (Soomro et al., 2023; Rems et al., 2024).

### 3.2 Structured materials and emerging porous

Because of their high reduction capacity and rich surface chemistry, two-dimensional materials such as MXenes (e.g., Ti_3_C_2_T_x_) have demonstrated outstanding potential for the adsorption of radionuclides and heavy metals (Amani et al., 2025a; Amani et al., 2025b). Nevertheless, a major obstacle is that they easily undergo oxidation when exposed to water (Iqbal et al., 2021). As far as adsorbents go, Covalent Organic Frameworks (COFs) are at the top of the food chain, with their crystallinity organized pores allowing for size-exclusion and targeted host-guest interactions (Aldana et al., 2024; Zhang C. et al., 2024). Despite advancements in stability and affordability, Metal-Organic Frameworks (COFs) are still mostly used in laboratory experiments. Instead, it is difficult to manage the brine waste that is produced, but next-generation ion exchange resins with improved selectivity and capacity are still the preferred technique for anionic contaminants, especially PFAS, at full scale (Elan Solan et al., 2025; Liu et al., 2025).

Carbonaceous, structured, and developing porous materials have great adsorption potential, but performance trade-offs must be considered. Sustainable carbonaceous adsorbents are cheap and eco-friendly, but their heterogeneous surface chemistry makes regeneration difficult and energy-intensive. MOFs, COFs, and customized composites have regulated porosity and specific functionalization, but their high synthesis cost and low stability in real-water matrices render them unscalable. In controlled lab contexts, novel porous materials perform well, but few studies have investigated their long-term durability, fouling resistance, or performance in complicated pollutant combinations. The gaps show that adsorbent class selection is situational. Real-world comparisons and scalable production should guide future research.

## 4 The optimal combination of adsorption and AOPs

**FIGURE 1:**
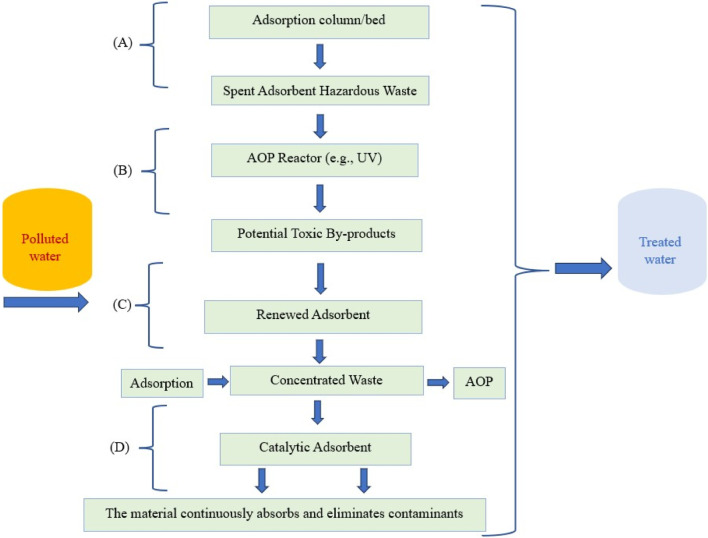
Comparison of water remediation adsorption-AOP integration diagrams with **(A)** Independent Adsorption Process, **(B)** Standalone AOP, **(C)** Sequential Integration (Destroying and Concentration), and **(D)** Catalytic Hybrid (Simultaneous Process).

Intentionally combining AOPs with adsorption creates a more effective treatment system than using each method independently. Table 1 analyzes the two approaches, demonstrating how they might complement one another to improve treatment results while mitigating their respective shortcomings. Moreover, Figure 1 illustrates this concept by depicting the progression from partially integrated systems to fully integrated ones. Below is a brief overview of the possible operating paths (A-D).

- A: Using adsorption alone effectively captures pollutants but requires the safe disposal of the resulting polluted waste adsorbent.
- B: The option of using AOP alone can degrade pollutants; however, it may not perform as well at low contaminant concentrations and can generate undesirable by-products.
- C: Combination in a sequential manner. The first phase, adsorption, concentrates the pollutants; the second phase, AOP, regenerates the adsorbent; and the third phase integrates both methods to achieve significantly greater efficiency.
- D: A catalytic hybrid system allows for the simultaneous absorption and breakdown of pollutants, minimizing waste as a single substance performs both adsorption and degradation.

**TABLE 1:** Modern AOPs and adsorption methods for water purification: a systematic review and comparison.

Technology category	Specific process/Material	Fundamental mechanism	Primary target pollutants	Advantages & recent innovations	Limitations and challenges	TRL and scalability considerations
AOPs	UV-LED/VUV AOPs	LEDs produce *OH radicals by directly photolyzing H_2_O_2_, O_3_, or H_2_O with ultraviolet (UV) or vacuum ultraviolet (VUV) light (Li et al., 2019a; Zhi, 2024)	Some short-chain PFAS, broad-spectrum organics, and NDMA (Darandale et al., 2023)	Potential for wavelength-specific activation; small, modular reactor designs; elimination of chemical sludge; and superior electrical efficiency and longevity of LEDs (Pattnaik et al., 2025)	Challenges with light transmission in murky water, accumulation of debris on quartz sleeves and LED surfaces, and tailoring UV light to target specific radical pathways energy expenditure for treatment of large volumes (Salve et al., 2022)	Showing that systems at scales ranging from bench to pilot are feasible. To avoid efficiency losses during large-scale deployment, reactor designs must take into account factors like light penetration depth, thermal management, and the spacing of LEDs. This will ensure that the reactor is lit uniformly and that there is enough photon flux throughout the entire volume
	Persulfate-Based AOPs	The process of producing SO_4_^−^ radicals involve activating persulfate (PS) or peroxymonosulfate (PMS) using heat, ultraviolet light, transition metals, or carbon catalysts (Wang and Wang, 2018; Xia et al., 2020)	Organic compounds that refuse to break down (such as PFAS, certain herbicides, and medicines) and which are effective across a broader pH range than *OH (Lee and von Gunten, 2023; Tian et al., 2022)	features an increased selectivity for organic molecules rich in electrons, a longer lifetime, and the possibility of activation without metals (via designed biochar or graphene, for example,) as well as non-radical routes (O_2_, electron transfer) providing a novel mechanism (Gao et al., 2022; Wang et al., 2021a)	The formation of toxic halogenated compounds (like bromate) in bromide-rich water sources; the potential for catalyst leaching in non-uniform systems; complex impacts on reaction matrices; the cost and handling of oxidants (Zhang et al., 2024a; Zhi et al., 2021)	Heterogeneous AOP catalysts at TRL 3–4 show lab-scale activity and selectivity. To ensure treatment performance and regulatory compliance at pilot or industrial scale (TRL 5–6), catalysts must maintain >90% activity over multiple cycles under variable pH, temperature, and contaminant loads, and robust strategies for capturing or neutralizing potentially toxic by-products are needed
	Heterogeneous Photocatalysis	Light-sensitive semiconductors (g-C_3_N_4_, doped TiO_2_, Bi-based) absorb light and produce electron-hole pairs (e^−^/h^+^) that initiate redox processes, leading to the formation of reactive oxygen species (ROS) (Ahmed et al., 2024; Wei et al., 2021)	Antibiotics, dyes, PPCPs, phenolic compounds (Vinayagam et al., 2024)	Exploring single-atom catalysts for maximum atom efficiency; developing S-scheme and Z-scheme heterojunctions for enhanced charge separation; utilizing the solar spectra via visible-light catalysts (Fang and Wang, 2023; Yi et al., 2024)	Reactor design for effective light harvesting; low quantum yield; rapid recombination of photogenerated charges; difficulty in recovering or separating catalysts in slurry systems (Li et al., 2021)	Stable, immobilized catalyst films or magnetically recoverable catalysts (TRL 4–6) are needed for pilot and industrial AOP systems. Catalysts must resist leaching, retain high activity across cycles, and separate easily from treated water to perform well and minimize operational costs at bigger scales
	Fenton-like Processes	Surface radical generation occurs when solid catalysts (Fe-, Mn-, Cu-oxides, zero-valent iron, etc.) heterogeneously activate H_2_O_2_ or PS (Li et al., 2019b; Lin et al., 2022)	Industrial effluent with a high concentration of chemicals, sludge dewatering (Wang et al., 2022)	Using waste-derived catalysts (such as slag or fly ash) and increasing stability with bimetallic oxides are two ways in which heterogeneous catalysts work at near-neutral pH. Heterogeneous catalysts also help to prevent the development of iron sludge (Montoya-Bautista et al., 2019; Yao et al., 2022)	Deactivation of the catalyst with time; possibility of metal leaching; consumption of H_2_O_2_ that does not produce any useful byproducts; Porous catalysts and their restrictions on mass transfer (Anekwe and Isa, 2025)	Works as intended for industrial effluent; current studies are concentrating on how long catalysts last and how to apply them to municipal streams
Adsorption Technologies	Engineered Biochar	Organic matter that has been pyrolyzed and then activated or doped with nitrogen, sulfur, or metals to achieve a specific porosity and surface chemistry (Blas et al., 2024)	Drugs, dyes, heavy metals (Pb^2+^, Cd^2+^), and, more PFAS through targeted design (Kayani et al., 2025; Parker et al., 2023)	“Designer” biochars, which are a byproduct of agricultural waste valorization and can display intrinsic catalytic capabilities for AOPs, have customizable surface functionality for targeted adsorption extremely affordable (Mulabagal et al., 2025; Sharma et al., 2025)	There is a trade-off between surface functional groups and porosity, performance can vary from batch to batch, and regeneration techniques are still in their early stages. The surface area is typically lower than that of activated carbon (Vedenyapina et al., 2024)	These technologies are versatile and durable, but widespread deployment (TRL 5–6) requires standardized production methods and strict quality control to ensure performance, dependability, and safety across operational scales
	MXenes and 2D Materials	Rich surface terminations (-O, -OH, -F) on 2D transition metal carbides/nitrides allow for intercalation and high adsorption capacity (Amani et al., 2025a; VahidMohammadi et al., 2021)	Radionuclides, dyes, heavy metals (Pb^2+^, Cr^6+^), and new uses for organic compounds (Amani et al., 2025b)	Impressive metallic conductivity and ultra-high surface area; adjustable interlayer spacing for ion sieving; easy functionalization; and promising electrochemical regeneration capabilities (Mishra et al., 2025)	Issues with oxidative degradation, particularly in aerated water, as well as the complexity and expense of large-scale synthesis and the lack of evidence about long-term stability in actual water matrices (Iqbal et al., 2021)	Materials stability and mass manufacturing cost hinder this technology’s advancement (TRL 3–5). These issues must be solved to maintain performance, make it reproducible, and make it practical for pilot or industrial use
	Covalent Organic Frameworks (COFs)	Crystalline porous polymers featuring rigidly pre-designed structures and micro-perforated pore openings (Akhtar et al., 2024; Zhang et al., 2024b)	Targeted micropollutants by gas separation, size-exclusion chromatography, and functional group interactions (Belete et al., 2023)	Highly selective, programmable, hydrothermally and chemically stable (unlike many MOFs); some even show photocatalytic activity (Aldana et al., 2024; Altmann et al., 2014)	Regeneration can be difficult for strong chemisorption; there are few studies on the long-term stability of complicated actual wastewater; and the expense of large-scale synthesis and purification is high (Satyam and Patra, 2024)	This technique is largely in materials discovery (TRL 2–3). Practical water treatment applications need reducing production costs and creating scalable synthesis procedures without compromising material performance or stability
	Next-Gen Ion Exchange Resins	Reversible exchange with dissolved anions or cations is a property of synthetic polymers that contain functional groups, such as amines (Froidevaux et al., 2016; Marin et al., 2024)	PFAS nitrate, perchlorate, arsenate, and many types of chains (both long and short) (Liu et al., 2025; Vakili et al., 2024)	Renewable, commercially available, highly selective for anionic PFAS (even at ng/L levels), and effective even when dissolved organic matter is present (Elan Solan et al., 2025; Wang et al., 2021b)	Background ion competition (SO_4_^2^, Cl^−^) decreases efficiency and speeds up saturation. a concentrated brine waste stream that needs management due to expensive regeneration; organic material fouling resin (Liu et al., 2022)	This technology is currently developing and requires specific knowledge (TRL 2–3). Scalable synthesis, real-world performance validation, and operational procedure standardization are needed to spread new resins for enhanced brine and short-chain PFAS treatment
Integrated & Hybrid Systems	Catalytic Adsorbents An exciting new area of study which is based on the creation and integration of sustainable and novel adsorbent and catalyst materials, such as biochar-based adsorbents, green nanoparticles, and biowaste-based catalysts Chitraningrum et al., 2023	One substance that incorporates a photocatalyst, scattered catalytic sites (such as Fe, Mn, or Co), and a high-surface-area adsorbent (carbon black, biochar, etc.) (Arabzadeh Nosratabad et al., 2024; Gu et al., 2024)	Combined contaminants, such as organics and heavy metals, or organics with a wide molecular spectrum (Chang et al., 2025)	A “self-cleaning” technique allows for material reuse; synergistic effects can improve both adsorption and catalysis; concurrent adsorption and *in situ* degradation prevent adsorbent saturation; and so on (Sun et al., 2025; Wu et al., 2024)	Difficulty in synthesizing; possibility of catalyst leaching; need for equilibrium between adsorption and degradation kinetics; stability when subjected to repeated cycles over an extended period of time. (Villa et al., 2021)	This new research area (TRL 2–3) may yield eco-friendly treatment medium. Before going to market, research must show that the materials are stable, scalable, and work well in real water matrices
	Sequential Concentration-Destruction	An AOP treats the concentrated regenerant stream after a selective adsorbent reduces the volume of contaminants from a large volume in a process train (Andreozzi et al., 1999; Hübner et al., 2024)	PFAS and other trace-level, high-priority pollutants in huge amounts of water (Adewuyi and Li, 2024)	By treating a small, concentrated volume, it dramatically increases the energy and cost efficiency of AOPs. The AOP phase can also replenish the adsorbent, finishing the loop (Cai et al., 2019)	Manage the concentrated AOP waste stream after incomplete regeneration, choose suitable adsorbent-AOP pairings, and apply more complicated process controls (Ponnusami et al., 2023)	Getting a lot of traction, especially when it comes to PFAS degradation, where concentration is key for practical economics

TRL, typical technology readiness level.

Carbonaceous adsorbents combined with photocatalysts form a hybrid material that improves pollution capture and degradation, according to recent studies. In the case of antibiotics like tetracycline, for instance, TiO_2_-biochar composites have been shown to achieve high adsorption and then undergo photocatalytic mineralization when exposed to light (Liu et al., 2024). According to the Biochar-TiO_2_ Review (2021), biochar-supported TiO_2_ has been found to degrade sulfamethoxazole in a similar fashion, with the carbon matrix concentrating contaminants on the catalyst surface and encouraging the production of reactive species. These examples highlight the potential of catalytic hybrid adsorbents for enhanced water treatment, since they can efficiently combine adsorption with photocatalytic regeneration.

Figure 1 shows how water treatment has progressed from using separate processes like adsorption and AOPs to more modern, integrated systems that remove contaminants in a more sustainable, efficient, and mutually beneficial way.

### 4.1 Concentration-decay loops in sequence

This approach entails concentrating contaminants from a significant amount of water using a selective adsorbent, such as an ion-exchange resin for per- and polyfluoroalkyl substances or a polymer for a particular drug (Adewuyi and Li, 2024; Hübner et al., 2024). The small-volume regenerant stream produced by an AOP has a high concentration, making radical-based destruction an efficient and economically viable option due to the high concentration of contaminants (Cai et al., 2019). Importantly, AOPs can be engineered to replenish adsorbent material *in situ* in addition to destroying pollutants, resulting in a closed-loop system that reduces chemical consumption and waste (Zhai et al., 2023).

### 4.2 Catalytic adsorbents with multiple uses

Here, the two roles are combined into one substance. The process involves functionalizing a porous adsorbent with catalytic nanoparticles, such as nano zero-valent iron, Fe_2_O_3_, or TiO_2_ (Gu et al., 2024; Sun et al., 2025). As a first step, this hybrid material uses adsorption to bring the contaminants to the surface. Next, the adsorbed contaminants are directly degraded at the surface when an oxidant (H_2_O_2_, persulfate) or light is introduced to the catalytic sites, which create radicals. A wasted adsorbent waste stream is eliminated and the material’s lifespan is greatly extended by this “self-cleaning” mechanism, which constantly regenerates the adsorption sites (Wu et al., 2024). Consequently, the surface concentration allows for more frequent radical-pollutant contacts, which in turn leads to improved performance due to the synergy.

Adsorption and AOPs have incredible synergistic benefits; however, several challenges remain. For instance, catalytic adsorbents show promising TRLs, yet their scale-up is limited by stability, regeneration, and leaching concerns. Meanwhile, concentration–decay cycles can improve removal efficiency, but they require precise control to prevent intermediate accumulation. Overall, hybrid systems hold significant potential; nevertheless, they need longer catalyst lifespans, seamless integration, and validation under real-world conditions.

## 5 Conclusion and perspectives

Rapidly eliminating diverse and persistent micropollutants, with significantly higher removal efficiency than either method alone, is achieved by integrating adsorption with AOPs. Separation and concentration using adsorption is an effective method, although AOPs are harmful. The contrasts and complementarities of technologies are compared in Table 1.

A noticeable trend is the smart integration of several technologies in water treatment. Synergistic systems of sequential process machines, as well as multifunctional materials present viable and sustainable solutions. They are able to eliminate problems of the saturated adsorbents waste and high energy consumption in AOPs that unsolved by the standalone systems. Here are the critical spaces where the next wave of innovation will be defined: “Material science” concerns how to make a versatile, stable, and selective material that is resilient in an industrial process.

A “versatile, stable, and selective” material performs reliably under realistic wastewater conditions in adsorption AOP hybrid systems. It should resist natural organic matter fouling, preserve structural and catalytic integrity across pH ranges, and resist oxidative degradation during repeated cycles. Selectivity removes target micropollutants, and magnetic recovery and self-regeneration make operation easier. Robust, scalable hybrid treatment approaches require materials with these combined features.

Real-time hybrids used in the management of pollutant load and water quality, are increasingly based on enhanced process control and on machine learning to optimize hybrid systems. Integrated systems are able to close the circular economy material loops by cleaning water and harvesting nutrients, adsorbents, and catalysts. Life-Cycle Assessment is performed to compare advanced integrated systems and conventional treatments in terms of their performance.

On the other hand, there are still issues with scalability, stability, and the production of by-products, however the strengths of adsorption and AOPs complement each other. Hybrid systems have great synergistic potential, but they need long-lasting catalysts, perfect integration, and validation in actual water. Scalable, regenerative materials and efficient reactor designs should be the focus of future research. Technology adoption and long-term water treatment solutions can be better guided by comparative studies conducted under realistic conditions.
